# Supplementary material for: Remote immune processes revealed by immune-derived circulating cell-free DNA
Source: eLife. 2021 Nov 29;10:e70520. doi: 10.7554/eLife.70520 (PMC8651286; doi:10.7554/eLife.70520)
Supplement: Supplementary file 2. [file elife-70520-supp2.docx]

**Supplementary File 1**:

| **Marker** | **Coordinate of central CpG** | **Number of unmethylated CpGs in block** | **Forward primer** | **Reverse primer** |
| --- | --- | --- | --- | --- |
| **Neut1** | chr9: 129648322 | All CpGs in block are unmethylated | TTTTAAGAAGTTTTTGTGTTATTAT | TCTAAAAATACCTAAATACAAACC |
| **Neut2** | chr8: 142180109 | All CpGs in block are unmethylated | GTTTGTTTTGAGATGTGAGAAT | ATAACATCCTTACAAACTCACAA |
| **Neut3** | chr11: 33308345 | 1 CpG in block can be unmethlated | TGTAGGTATTTTAGATTGGGG | AATTATCCAACTCCTCACTCTTA |
| **Mono1** | chr11:32055233 | 1 CpG in block can be unmethlated | TTTGTTAGGTTAAGTAATTTGTAAA | CATCTCCTACTTAAATAACTTCAAT |
| **Mono2** | chr10:114911652 | All CpGs in block are unmethylated | TGAAGGAAATGAGAGTAAAGGT | CCCTTCTCCCTAAAAAAAAC |
| **B-cell1** | chr11:121440880 | All CpGs in block are unmethylated | AGGTTGTTTTTTTATTTTTTAGAT | TTTCCCTCCCTTTAATAACTAT |
| **B-cell2** | chr17:3493666 | All CpGs in block are unmethylated | TTTTAAAGAAGTTTTTATGGGT | ATAAACCAAACAACACTACACAT |
| **B-cell3** | chr11:34167855 | All CpGs in block are unmethylated | ATTTTTTTTGGTTGGATTGTT | TCACAAACACACAAACCCAA |
| **T-cell1** | chr14:61801201 | All CpGs in block are unmethylated | GGTGTTATAGGTAGGGTAGAGAA | CCAACATTTATCATTTTCTTCA |
| **T-cell2** | chr13:24825973 | All CpGs in block are unmethylated | AGTATTTTTATTGGGTTGGAT | CCTACTACCTCAAATTAACTAAAA |
| **CD8A** | chr2:87012810 | All CpGs in block are unmethylated | TTAGTTTTTTTAGTATGATTTTGAG | CACCACAAAAATCACAATACTAT |
| **CD8B** | chr:87048747 | All CpGs in block are unmethylated | GTTAAGAAATTAATAGGAAAAAGAA | AAAACCCCATATTACTTCCC |
| **TREG1** | chrx:49118313 | All CpGs in block are unmethylated | TTAGGTTTGGATTTTAATTTTG | CCCTAACCCTTATCTACTCCA |
| **TREG2** | chrx:49117224 | All CpGs in block are unmethylated | TGGGTTTTGTTGTTATAGTTTT | ATATCTACCCTCTTCTCTTCCTC |
| **EOSI1** | chr3:195974300 | 1 CpG in block can be unmethlated | GGGGTATTTTTTATTATTTTATT | ACACACAACTTCAAAAACTTCA |
| **EOSI2** | chr4:3123132 | All CpGs in block are unmethylated | GGAGTTGTTGTAGTAGTTTTTTAGA | AAATTCCACAATACTCCCACTA |
| **EOSI3** | chr1:6341327 | All CpGs in block are unmethylated | TTTGAGAGTTGTTTATAATAGGGT | CCTCCCTTCTCCACAAACTA |
